# University of Maryland

**Published:** 1844-09

**Authors:** 


*University of Maryland.*—The medical department of this institution, we find from their annual announcement—opens this year with high prospects and increased facilities of medical instruction.

The vacant chair of theory and practice of medicine is now permanently and satisfactorily filled by Elisha Bartlett, M. D., late Professor of Theory and Practice of Medicine, in Transylvania University.

Lectures on Practical Pharmacy will be given by David Stewart, M. D. professor of this branch in the Maryland College of Pharmacy.

Arrangements also have been made for opening a Medical Reading Room in the College, for the benefit of the students.

The lectures for the ensuing winter begin last Monday of October and continue till first of March.

